# Between stigma, misinformation and delay of diagnosis: healthcare worker’s perspectives on leprosy care in Sindh, Pakistan

**DOI:** 10.1186/s12879-026-12551-z

**Published:** 2026-02-02

**Authors:** Sophie C. W. Unterkircher, Abdul Salam, Isabel Fernandes, Anil Fastenau

**Affiliations:** 1 Department of Global Health, Institute of Public Health and Nursing Research, University of Bremen, Universitätsallee 1B, 28359 Bremen, Germany; 2 Marie Adelaide Leprosy Center (MALC), Mariam Manzil, A.M. 21, Off Shahrah-e-Liaquat, Saddar, Karachi, 74400 Pakistan; 3 German Leprosy and Tuberculosis Relief Association (GLRA/DAHW), HQ, Raiffeisenstraße 3, 97080 Wuerzburg, Germany

**Keywords:** Leprosy, Neglected tropical diseases, Stigma, Discrimination, Diagnostic delay, Misinformation, Healthcare, Pakistan, Stigma reduction, Low endemic

## Abstract

**Background:**

Beyond the physical symptoms of leprosy, persons affected by leprosy (PAL) continue to face stigma and discrimination, also in the low-endemic setting of Pakistan. Leprosy-related stigma and misinformation impacts treatment adherence and health-seeking behavior of PAL, ultimately increasing risks of disability and increased disease transmission. Healthcare workers (HCW) play a pivotal role in detecting, diagnosing, counseling and supporting PAL. Their insights are essential in developing effective interventions to combat the stigma and improve the overall well-being and confidence in healthcare of those affected.

**Methods:**

Twenty-one in-depth individual interviews were conducted with dermatologists, dermatology residents, general physicians, leprosy technicians and nurses. A combined inductive-deductive thematic analysis approach was applied based on a “conceptual framework of the dimensions of stigma, its manifestations and impact on health outcomes” developed by Mukerji and Turan.

**Results:**

The study revealed that stigma and misinformation were shaped by contextual factors, including the absence of a national leprosy program, limited public awareness, patriarchal gender norms and socio-economic disadvantages. HCW reported frequent delays in diagnosis, psychological distress, limited awareness and socio-economic burden due to leprosy and its stigma among PAL. Although HCW demonstrated strong medical knowledge, low levels of leprosy exposure outside specialized centers reduced diagnostic confidence of non-dermatologists. Trust-building, psychosocial counseling and sensitive handling of disclosure proved essential for treatment adherence. Gender dynamics also affected care: women were more likely to hide symptoms due to the anticipated stigma, whereas men were more likely to comprise treatment adherence due to economic responsibilities.

**Conclusions:**

In low-endemic settings like Pakistan, stigma, misinformation, and structural barriers intersect to drive diagnostic delay and limited access to care. Strengthening leprosy care requires (1) Increasing public awareness and reaching broader audiences through social media and digital health tools (2) collaboration with local and alternative medicine providers to reduce misdiagnosis (3) targeted training for HCW on psychosocial counselling and gender responsive care; and (4) evaluating and improving care interventions through close collaboration with PAL and community leaders. Further, integrating leprosy control into national programs and mental health services would improve comprehensive care and support stigma reduction.

**Clinical trial number:**

Not applicable.

**Supplementary Information:**

The online version contains supplementary material available at 10.1186/s12879-026-12551-z.

## Background

With more than 200 000 new cases reported annually, leprosy remains an endemic disease in more than 120 countries [1]. As a major contributor to preventable disability, leprosy is a chronic infectious disease caused by Mycobacterium leprae. Infection transmitted through prolonged contact with an untreated leprosy patient can result in symptoms affecting the skin, peripheral nerves, upper respiratory tract mucosa and eyes [2, 3]. Onset of symptoms can take up to twenty years, making it difficult to detect and treat the disease with the recommended multi-drug therapy (MDT) early on [3]. In addition to physical symptoms, people affected by leprosy (PAL) experience discrimination and stigma, which ultimately affects their mental health and overall quality of life. Although leprosy is curable and great strides have been made in reducing the global burden of disease, the stigma associated with the disease has persisted since the middle ages [4].

”The figure of the lepers has remained for centuries a symbol of the worst that God could visit on humanity” [4]. For centuries, people-affected would be declared as unclean or already dead, thus leprosy ultimately became a global synonym for exclusion [5]. Stigma has been defined as „a social process or related personal experience characterized by exclusion, rejection, blame, or devaluation that results from experience or reasonable anticipation of an adverse social judgement about a person or group identified with a particular problem.” p.536 [6]. To conceptualize stigma in this study, four dimensions of Mukerji & Turan’s framework were explored: anticipated, perceived, internalized and enacted stigma [7].

Studies have shown that stigma, often enforced by misinformation, can be a deterrent to care-seeking, particularly in places with few leprosy cases [8, 9]. The lower-middle-income country Pakistan exemplifies such a context. It can be classified as a low endemic setting, with approximately 58,500 registered cases in 2020 and 200–300 new cases each year, and is thus on the way toward elimination [10]. However, there are limited studies exploring leprosy related stigma in Pakistan, some dating back to thirty-six years ago [11]. Furthermore, current research mainly entails evidence on interventions that were designed for high-endemic settings [12]. Yet, they prove to be unfit for low-endemic settings, like Pakistan, where leprosy has officially been declared eliminated as a public health issue since 1996 [13]. Several factors contribute to this inapplicability, such as cost-ineffectiveness, limited expertise and capacity due to disease rarity, differing transmission dynamics and distinct patterns of internal and external stigma [14–16]. As identified by two systematic reviews on case detection methods and stigma reduction interventions, no literature was found for Pakistan, thus underlining the narrative of this paper. Therefore, this study aims to investigate stigma and its relationship with misinformation and diagnostic delay, in a low-endemic setting. The case study is Sindh, a province in Pakistan with the highest leprosy burden despite the country’s overall low prevalence [17].

We will dive into the perceptions and work experiences shared by dermatologists, residents specializing in dermatology, nurses, leprosy technicians (LTs) and general physicians (GPs), practicing in the field of leprosy in Sindh, Pakistan. Due to their central role in diagnosing and treating PAL they take on many influential roles, not only as care providers, but also as confidants, opinion-leaders and counselors. Therefore, it is critical to examine whether stigma and misinformation among healthcare workers (HCW) persists, as suggested by previous studies [17–19]. Moreover, it is also important to explore the HCW-PAL relationship, as this can strongly affect care-seeking and treatment adherence among PAL [17–19]. Literature has shown that interactions with HCW can impact PAL’s understanding and self-perception of leprosy in both positive and negative ways [17–19]. These interactions, therefore, play a pivotal role in shaping the overall experience for those living with leprosy. Thus, HCW’ insights are invaluable for informing policy and practice recommendations aimed at improving the mental wellbeing of PAL, ensuring equitable and high-quality access to health services and shaping community perceptions and awareness of leprosy.

## Methods

### Study design

This study employed a qualitative descriptive design, informed by phenomenological principles (Fig. 1, Supplementary Material 1). It explored HCW’s work experiences, personal perspectives and reflections on providing care to PAL and associated stigma. To contextualize stigma, four overarching types of stigma defined by Mukerji & Turan are considered [7]: (1) Anticipated stigma: “expectations of discrimination, stereotyping, and/or prejudice from others post disclosure due to a stigmatized condition or behavior” (2) Perceived: “feeling that those without the condition perceive people with the condition negatively” (3) Enacted: experiences of discrimination, stereotyping, and/or prejudice from others in the past or present due to a stigmatizing condition or behavior” (4) Internalized stigma: “endorsing negative feelings and beliefs associated with the stigmatized condition and applying them to the self”. As this study focused on the perceptions of HCW, enacted and internalized stigma will be displayed solely from an observer perspective. To further illustrate the many facets of leprosy related stigma, a version of the “Conceptual Framework of the dimensions of stigma, its manifestations and impact on health outcomes” developed by Mukerji and Turan [7] was adapted to the study’s context. Although originally developed for tuberculosis-related stigma in India, this framework was considered appropriate, as both conditions are chronic infectious diseases, marked by social stigma. To account for the Pakistani context and leprosy-specific factors, an overarching category on the contextual factors was added. Furthermore, all categories were considered to have a mutual influence rather than being strictly sequential. The ‘consequences of stigma’ category was extended to include misinformation, reflecting the low endemic setting in Pakistan where a small number of cases lead to widespread misconceptions alongside stigma. At each stage of the research process, potential sources of selection, information and observer bias were systematically identified and addressed (Fig. 1; Supplementary Material 1).

### Study setting

The study focused on Sindh, the region surrounding the city of Karachi. Interviewees were working both in an outpatient and inpatient settings at private and public hospitals, coming from urban as well as surrounding rural areas. Sindh, the second largest province by population with 47.89 million as of 2017, lies in the southeastern region of the country [20] and reports the largest number of leprosy cases throughout the country, making up 38% of all cases as of 2023 [17]. The Marie Adelaide Leprosy Center (MALC), a non-governmental organization (NGO), based in Karachi, serves as the headquarters for national leprosy control activities and operates a large in-patient facility.

### Participants

Due to the low-endemicity of leprosy in Pakistan and the fact that HCW treating PAL are relatively specialized and networked, participants were identified using a mixed purposive and snowball sampling technique [21]. Snowball sampling was used to complement purposive recruitment, allowing initial participants to refer colleagues within their professional networks, given the limited number of HCWs with relevant experience. Eligible participants were HCW providing care to PAL, aged over 18 years, proficient in English or Urdu and able to provide written informed consent.

Sampling was structured into two phases: *The first phase* of recruitment and interviewing took place between May 5th and June 26th, 2024, and was limited to English-speaking dermatologists from a network of both private and public hospitals, who would be participating in online in-depth individual interviews (IDI). Upon request, two senior dermatologists had asked to be interviewed prior to sharing contacts of additional colleagues. Therefore, two pilot interviews were conducted before further participant referral continued.

With the help of two staff members at MALC, the research project was introduced to potential participants. Those who expressed interest were contacted via E-Mail and the communication platform Whats-app to provide further information. Emails included a detailed information sheet and consent form. In phase one, a total of 29 participants were contacted; two cancelled and 15 did not respond. If necessary, e-mails/ Whats-app messages were followed up weekly, up to three times.

*The second phase* of recruitment and interviewing was conducted on site at MALC from October 9th until October 18th, 2024, and extended to dermatologists, GPs, nurses and LTs, working in a private NGO hospital setting, speaking English. Everyone that was approached on site agreed to participate. Interview participants did not receive compensation.

### Data collection

Data were collected through-semi structured, in -depth individual interviews with two exceptions where participants preferred to be interviewed together. The interview guide (Supplementary Material 3) was developed based on existing quantitative stigma scales, adapted to the local context and pre-tested with field experts [22–24]. Following pilot interviews, the guide was refined to improve clarity and reduce social desirability bias. The interviews were conducted in English and averaged 35 minutes in duration. Two via the online conference platform Zoom, ten via telephone, and nine in person at MALC. All participants were informed about the study’s purpose, procedures, and their rights, and were asked to sign consent forms prior to the interview. Participation was voluntary, and participants could withdraw from the study at any time. Data collection continued until no new themes or concepts emerged, indicating that data saturation had been reached.

### Data analysis

Interviews were audio recorded and transcribed using the external artificial intelligence (AI) software noScribe. Transcripts were manually verified and then pseudonymized by SCWU. A combined deductive-inductive thematic analysis, guided Braun & Clarke [25] was applied to analyze collected interview data. Given the unique context of HCW experience in Sindh, inductive coding allowed for new patterns and context-specific themes to emerge. The first steps involved familiarization with the data and the development of a coding-scheme, based on Mukerji and Turan’s theoretical framework [7]. Coding was supported by the qualitative analysis software MAXQDA 2022. Codes were then refined inductively, and sub-categories were built. To ensure consistency and reduce potential bias, the analytical process included cross-checking codes among co-authors, maintaining reflexive journals to document analytic decisions, and iterative discussions to ensure transparency and rigor in theme development. Lastly, key-categories were summarized for each interview and organized into thematic tables. The inclusion of quotations support transparency and transferability of the interpreted findings.

### Ethical approval

Ethical approval was obtained from both the Ethical Review Committee of MALC (MAC/ERC/2024/01) in February 2024 and of the University of Bremen (2024-10) in April 2024. Research was conducted in accordance with the Declaration of Helsinki. All data is pseudonymized and stored on a secure, password protected device and will be deleted after five years.

## Results

### Participant characteristics

In total, 21 HCW participated in the interviews, with an average age of thirty to forty years. The majority of HCW were female and had between one to ten years of work experience. Half of the participants were consultant dermatologists and dermatology residents, while the other half comprised nurses, LTs and GPs (Table 1).

**Table 1:** Participant characteristics

Variables		Number of Participants (*N* = 21)
Age	20–30	3
	30–40	11
	40–50	4
	> 60	1
	Unknown	2
Sex	Female	16
	Male	5
Profession	Consultant Dermatologist	4
	Consultant Dermatologist involved in medical education	2
	Dermatology Resident	7
	General Physician	2
	Nurse	4
	Leprosy Technician	2
Years of experience	1–5 years	6
	5–10 years	6
	10–15 years	2
	15–20 years	3
	> 30 years	1
	Unknown	3

**Fig. 2:**
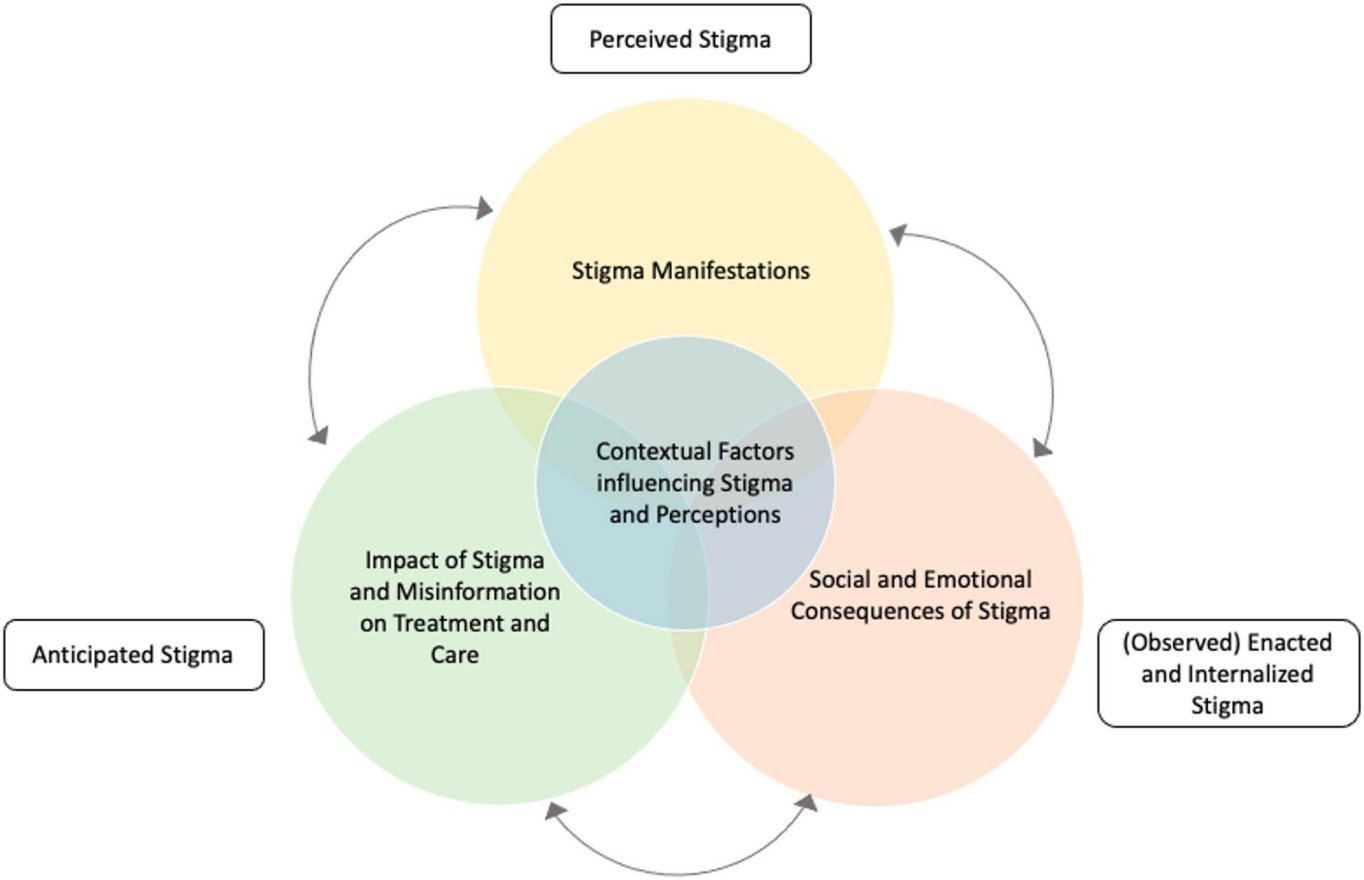
Interconnected pathways of stigma. Based on Mukerji and Turan’s framework [9] to illustrate the dimensions of stigma, highlighting the interplay between manifestations, consequences and contextual factors shaping them

Based on the collected interview data, four themes emerged along with respective sub-themes: (1) Contextual Factors Influencing Stigma and Perceptions (2) Stigma Manifestations (3) Social and Emotional Consequences of Stigma (4) Impact of Stigma and Misinformation on Treatment and Care. Mapping these themes onto the conceptual framework of stigma dimensions by Mukerji and Turan [7], key findings align with the categories of anticipated, perceived, (observed) internalized and enacted stigma (Fig. 2).

### Contextual factors shaping stigma and perceptions

All identified key themes are embedded within the broader contextual factors shaping leprosy-related health services in Sindh, Pakistan, specifically the treatment process, medical education and societal understandings of leprosy.

#### Leprosy health services

While MDT is free of cost throughout the whole country, there is no official national leprosy control program in Pakistan. Control activities are mainly provided by the two civil society organizations, Aid to Leprosy (ALP) and MALC. These NGOs offer free consultations, medications and medical treatment. Additionally, PAL referred to general hospitals through MALC receive discounts or free consultations. With 157 control centers operating in Sindh, KPK, Balochistan, Gilgit Baltistan and Azad Kashmir, a proper organized system of case documentation helps HCW with the screening, monitoring of treatment and follow-up of PAL and their household contacts. Next to that, both NGOs started with the implementation of Single-Dose-Rifampicin Post-Exposure-Prophylaxis (SDR-PEP) among contacts of PAL in 2022. Furthermore, interviewees shared that MALC organizes screening and educational camps in a variety of settings, such as churches, madrasas (educational institutions), councilor offices and private clinics. ALP and MALC additionally provide educational support, vocational skills programs and social work such as marriage counseling and matchmaking.

She [Dr. Ruth Pfau] had a bigger picture for leprosy. She used to see a patient as a whole. (Dermatologist, 47, Interview 13 – talking about the founder of MALC)

#### Treatment procedure

Dermatologists working in public healthcare centers explained that patients initially visited the outpatient department (OPD) of their hospitals, or were directly referred by general practitioners, then a clinical diagnosis followed a slit-skin smear test. After a diagnosis was made, PAL were registered with MALC for follow-up purposes and referred to one of their centers for confirmation of diagnosis, further counseling, treatment and support.

But we entertain such patients not for a long time. We examine and diagnose the patient and then we refer them, so they spend most of their time in MALC. […] They open up to them more than us. (Dermatology Resident, 28, Interview 6)

Although most counseling occurs at MALC, one dermatologist working in a general hospital explained that the Dermatology Life Quality Index Score (DLQI) is applied to assess the mental health and stress levels of PAL, helping to determine whether psychological support is needed. Furthermore, beliefs of PAL are addressed, hope is fostered, and knowledge is shared.

So, my big approach starts always [with] counseling, from telling them what they have, to about treatments, to telling them how we will be dealing with them in the future and how they can reach out to us in different sort of things. (Dermatologist, 33, Interview 11)

As PAL often find themselves in a stressful and fearful state of mind, one dermatologist shared his way of providing support:

So, the first thing I try to do is to laugh off the stress that they have and that we as health workers can have. [I] tell them that it’s like any other disease and that we are sitting in a room which can have 100,000 germs, bacteria in it, or viruses, which we cannot see, but they are entering our body and we can get any disease. […] I always give them my example that I’m taking medicines for my blood pressure and for my heart, which I have to continue until I go to my grave. But for you, if you start your treatment, it is time limited and it’s free. (Dermatologist, > 60, Interview 2)

Next to addressing potential misinformation related to the origins of the disease and other concepts, a significant aspect of counseling involves acknowledging and respecting the religious and spiritual beliefs of PAL. It can pose as a coping mechanism and foster a sense of hope.

[…] Pakistan is, I would say, very much a religious country, so most people are quite spiritual. So, we had to attend to that, whatever religion they belong to. [We tell them] […] you are not alone, and your family is there to support. We are there to help you. […] God is there to help you. (Dermatologist, > 60, Interview 2)

#### Education and knowledge of leprosy

All HCW displayed medically informed knowledge regarding the pathogenesis, clinical considerations and treatment of leprosy. While dermatologists, dermatology residents, LTs and nurses affirmed the curability of the disease, a fact supported by evidence, two GPs interviewed maintained the belief that leprosy is incurable.

Actually, it doesn’t go away. The medicines we give to leprosy patients […] prevents further spreading of the disease. We can’t permanently cure it, but we can give them static ones. Most of the disease will not be cured. (GP, 25, Interview 15)

Moreover, two dermatology residents stated they had not learned about leprosy stigma during medical school so far.

No actually, I didn’t learn. […] I have listened to these stories from patients, not in education. In books, it’s just a disease, there is no stigma to it, but in general population, people have obviously stigma to it.” (Dermatology resident, 28, Interview 12)

As one interviewed professor explained, teaching about leprosy had only previously been included in the undergraduate level, yet more education is required for undergraduates and postgraduates, especially among GPs. With that, it has become mandatory for doctors specializing in dermatology to do two weeks of rotation in the field of leprosy. Due to the low endemicity of leprosy cases, such rotations mostly take part at MALC, as it was described to be very uncommon to see PAL outside of the environment of “those centers.”

See, when I came to MALC, I learned the process of early diagnosis, detecting leprosy, diagnosing leprosy, but when you are in the outside world practicing, the first thing is that obviously you should forget the disease. I read it in my textbooks, but I never ever had the experience to see and never ever had the courage to see the patient because it is not a common disease. (Dermatologist, 47, Interview 13)

Altogether, HCW expressed that while their education had provided them with a solid theoretical foundation of working in the field of leprosy, the reality of working as a nurse or doctor vastly differs. Hence, MALC was named as an exemplary model for teaching HCW about the many facets the field encompasses.

When we come out of the bachelor schools, we are like horses who have bought those helmets. We just see what we learn. But MALC removes that to see the bigger picture. […] MALC is about the bigger picture. (Dermatologist, 47, Interview 13)

#### Societal perceptions of leprosy

Although, participants noted a decline in leprosy cases and a reduction in associated stigma, a continued lack of awareness still shapes societal perceptions towards leprosy and the people affected by it.

This is the kind of disease, which is dying from the population, […] but it is also dying from people’s mind. (Dermatology resident, 28 years, Interview 3)

Thus, interviewees felt that limited public knowledge fuels misinformation and concerns. For instance, fears that leprosy remains highly contagious even after treatment, or beliefs that it is very “deadly” and caused by divine punishment or wrong doings. In line with that, HCW agreed that PAL suffer from a generally low status in society, often struggling to interact with the public due to discrimination, isolation and an overall negative social attitude. As a result, it is seemingly common for PAL to face hardship when trying to find someone in marriage, or finding/keeping a job.

In our society, people have really little knowledge regarding leprosy. The only information they have got is that it’s a really dangerous, contagious disease, and the people who contract it, they should be just secluded from the society.” (Dermatology resident, 28, Interview 3)

Several participants suggested that leprosy stigma appeared to be more prevalent in certain communities, particularly among Indian and Afghan immigrant communities (e.g., Hazara) compared to Sindhis and Baloch people.

### Stigma manifestations (Perceived stigma)

To acknowledge how attitudes of HCW are shaped, it is crucial to consider in which ways leprosy and stigma are perceived and understood by them.

#### Perceiving leprosy

While the perception of leprosy differed between HCW, LTs, nurses and dermatologists described leprosy to be more severe in contrast to other diseases. Being recognized as “more destroying” and “mentally draining”, leprosy was perceived to embody more than just a disease, as sudden deformity and disability can cause mental, social and economic hardships.

There are a variety of aspects to the disease, not just medical, but social, economic. And there is mental stress. (Dermatologist, > 60, Interview 2)

On the other hand, a few residents and dermatologists understood it to be like any other disease, without distinguishing variables beyond its pathology.

I mean we don’t see it beyond a disease. It’s a disease, and it will be cured. (Dermatology resident, 28, Interview 3)

#### Understanding of stigma

Overall, there was a noticeable level of awareness regarding stigma among HCW; however, most nurses encountered greater challenges in identifying what stigma entails.

HCW described leprosy stigma to be a reaction to the disease itself, to certain beliefs, the fear of disclosing one’s diagnosis and to the challenge of surviving in society, all characterized by exclusion and embarrassment. From their perception stigma is something that leads to depression, frustration, lack of confidence and isolation. One dermatologist cited that the disease itself is not associated with stigma, but the morbidities it can cause then turn into stigma. Many emphasized that visible disfigurement and disability contribute significantly to stigma, alongside misinformation and limited public knowledge.

Stigma means kalank. Kalank is something, bad reputation, which cannot be washed from you for lifelong. Kalank is something, it cannot go away from your life or from your personality. (Dermatologist, 30, Interview 5)

### Social and emotional consequences of experiences with leprosy and stigma

Fears and experiences of leprosy related stigma have both social and emotional consequences, impacting HCW socially and influencing their interactions with PAL. HCW shared observations of stigma enacted against PAL, the tendency of PAL to internalize stigma, and how these circumstances impact their own practice.

#### Social impacts on HCW

Although a handful of HCW have encountered stigma and suspicion by family and friends, that voiced concerns of maintaining a safe distance, fearful of disease transmission, it appeared to be the exception. Initial apprehension among friends and family was solved by addressing their assumptions.

[W]hy are you continuing to work over there? Why don’t you leave? Why don’t you do something else? But that was not very frequent. That was just occasional. (Dermatologist, > 60, Interview 2)

#### HCW-PAL relationship and disclosure challenges

In a general sense, it is stated that a trusting relationship is frequently established between HCW, and PAL. HCW tried to be forthcoming by making themselves “humble”, cooperative, and available to PAL, for them to feel comfortable. Due to the professionally required confidentiality, interviewees suggested that many PAL felt more secure in talking to them, rather than to their close friends.

They become just like families. You see such old, old patients they become fond of here. They go home for only one to two days [and] again, they will come here, [they] want to stay here (GP, 48, Interview 15)

Most nurses, LTs and dermatologists interviewed expressed a personal commitment to avoid misdiagnoses and wrong treatment. However, a senior dermatologist (Interview 2) recounted a contrasting viewpoint shared by a younger colleague, who stated: *“we are not interested in a disease*,* which does not give us costumers*,* […] it is not going to pay us anything.”*

Additionally, HCW explained that PAL were encouraged to share the news of their diagnosis with their most immediate families to establish a robust social support system. This support could be further enhanced by engaging with other PAL at MALC, as one of the doctors stated:

And we see the leprosy patients sitting beside them and they talk with them, and their tension is somewhat relieved. (GP, 25, Interview 15)

LTs have provided more insights into the complexities of disclosing a diagnosis to family members. They explained that while they sought permission from PAL to contact their families, a lot of convincing through counseling was usually required. This approach was defined as essential, not only for establishing a support system but also for facilitating screening efforts. LTs shared that, as of late, the exact location of a PAL is traced and registered to create a better case map. This ensures that all social contacts of PAL are screened and provided with SDR as post-exposure prophylaxis, to interrupt transmission and decrease the risk of new leprosy infections. However, this process could be particularly challenging when they tried to seek permission from women or communities that hesitated to cooperate in disclosing information about suspected cases. Therefore, priests, Maulanas and Qari Sahab’s were commonly approached as stakeholders to communicate with affected communities.

Some people have a treatment from leprosy, and they said no, no, there is no patient here. And he was a patient, but he said nobody is living in this house. (LT, 37, Interview 21)

Interviewees expressed that many PAL were worried to be disowned from their families, excluded from social gatherings, receive hatred or experience embarrassment. Therefore, LTs working in the field explained that sometimes they were asked not to officially visit the home of a PAL or park their official cars further away.

[S]he said if you are coming to me, so please just don’t disclose that you are coming from which area. So, I was there, and I was wearing this apron which is MALC was written. I took it off and I put it in my vehicle, and I asked driver to go back. I will walk to them. And then when I went there, then the sister-in-law of that lady, she came down and asked who is this lady. So, she said no, she is only coming for polio drops. Although the patient was discharged for about 15 to 20 years, but still they were hiding. And they said, you know, if my husband [has] some kind of ulcers, […] we don’t tell them that this is due to leprosy, we tell them it’s due to diabetes. (LT, 33, Interview 20)

HCW shared that in some cases PAL even refused to start treatment in order to hide their diagnosis from family and friends. On the other hand, some PAL easily told their family members and surroundings about having leprosy.

#### Witnessed behavior and reactions of PAL to their diagnosis (Observed enacted and internalized stigma)

##### Influence of background factors

Generally, participants stated that PAL presenting to them were usually middle aged (30–60 years), with only a few cases of affected children. Along with that HCW described leprosy as a “disease of poverty”, recounting that the majority of PAL belong to a lower socio-economic status (SES).

[T]hey live in a crowded space, 15 to 20 members in a single home. (Dermatology Resident, 32, Interview 4)

Although it is rare to encounter PAL with higher SES, HCW noted that counseling such individuals was more challenging, as they often either denied the diagnosis or questioned its treatability. An LT working in the field narrated:

Recently we just had a lady with leprosy, and she was from [a] very well-educated family. [She was] like, why is it happened to me? I am very clean. I have no one in my family that was untidy, like we don’t have this kind of germs and all. (LT, 33, Interview 20)

Another LT working in the field agreed, suggesting that stigma appeared to be more prevalent among people from higher SES groups compared to those from low SES backgrounds.

##### Gender differences

More than half of the participants stated that they had seen more male PAL than female PAL, while a few suggested that the ratio between both genders was equal.

Generally, females affected by leprosy were described to “suffer more”, to “have a lot of issues” since “women are blamed more in a male dominated society”. They were reported to fear disclosing their diagnosis to relatives and therefore tended to hide it.

Females suffer more. They hide their symptoms. […] They are afraid of getting divorced from their husband [and to] have to go back to their parents’ house. (Dermatologist, 38, Interview 1)

Unmarried women were also worried about how their appearance might be affected by scarring or hyperpigmentation, as this might affect their marriage prospects.

She was literally crying: “what I will do, how I will get married”, her mother was there asking: who will marry her? (Dermatology Resident, 32, Interview 4)

As women mostly stay at home, taking care of household chores and family matters in Pakistan’s cultural context, some had explained to HCW that they felt uncomfortable seeking medical care, especially if it required traveling to larger cities. Moreover, interviewees agreed that initial symptoms may not be caught, because they are often covered by culturally appropriate clothing. Men on the other hand were more likely to notice related symptoms, either in themselves or through their surroundings, because they traveled and worked more outside their home.

Males […] generally they reach out to us and they go to their doctors. Being an Islamic country, most of the females are not comfortable going out, reaching out to doctors [a]nd […] coming to cities. (Dermatologist, 33, Interview 11)

Although HCW reported that female PAL tended to show better treatment adherence once diagnosed, some also noted that women usually wanted to leave treatment early, to return home and fulfill household and family responsibilities. Male PAL, on the other hand, were described to be less compliant with treatment, as they were more likely to prioritize work schedules and duties. With men being mostly responsible for assuring economic stability for the family, they were said to experience a lot of pressure and to continue work regardless of their symptoms.

In our society, men have more responsibilities, like they have to do the economic thing, […] they are the breadwinner. […] Mostly they are under pressure of children. That if I am not going to work, I, rather than have the anesthetic hands and feet and the sores. (LT, 33, Interview 20)

##### Sense-making and beliefs of PAL

HCW encountered a variety of reactions when diagnosing people with leprosy, often driven by a lack of public knowledge or misinformation about the disease. While one GP shared that PAL were usually aware of leprosy and its contagiousness, but simply “*don’t care*” (Interview 15), most HCW suggested that the majority of PAL were unaware of their diagnosis and blamed themselves. In fact, the only information that many PAL recounted is that leprosy was “*very dangerous*” (Interview 3), contagious, and not treatable. Especially in the case of leprosy reactions (Type 1 or 2) re-appearing, PAL believed to be infected again. Some also perceived leprosy to be hereditary or a progressive disease like cancer.

[…] [T]hey think if people come to know about my disease, they will not come to my daughters for marriage. Because then they think, oh this will carry to my genes. (LT, 33, Interview 20)

It was commonly observed that PAL tended to come up with any kind of explanation, when trying to understand where leprosy came from and why they were affected. Hence, some PAL believed leprosy to be a curse. Reported causes included black magic, solar or lunar eclipse, sins, the evil eye, failure to follow religious command, personal wrongdoing, or negativity running in the family.

This person might have done something really wrong in their life or they might have done some heinous crime. So that is why God is punishing them for this. (Dermatologist, 30, Interview 3)

Furthermore, some PAL made sense of their leprosy infection by attributing it to being bitten by an animal or a mosquito, eating certain foods from specific areas or having fallen somewhere. Such beliefs were seen in both educated and non-educated PAL, as described by interviewees.

They are usually related to some black magic, in their family, and it’s a kind of myth. [They say: ] I have taken this food from that area. That’s why I got this disease, or they have some negativity in family running. Or at work place they said that person might have got some magic, that’s why I am suffering. (Dermatologist, 30, Interview 5)

Overall, PAL were described to be very disturbed and “mentally exhausted” when finding out about their leprosy diagnosis, suffering from low self-esteem, guilt, anxiety, shock and confusion.

Every time when she comes to me, in the initial stages […] she was just crying, mentally, like she was very disturbed. [Asking] why is it happening to me? (LT, 33, Interview 20)

Although in many instances they were thankful that they were “still better than those conditions they have seen on google” (Interview 20), they expressed a variety of fears. Interviewees shared that some started isolating themselves, while others would not be able to return home after treatment, and therefore started working at MALC as “ward boys” or “serving tea”.

### Impact of misinformation and stigma on treatment and care (Anticipated stigma)

As indicated above, several consequences arising from fears and experiences of stigma affect both HCW and PAL emotionally and socially. Additionally, the following paragraph focuses on how stigma and misinformation affect the operational aspects of healthcare delivery and how it can be overcome.

#### Hesitancy to treat PAL

Interviewees agreed that especially during medical school, dermatology students were often reluctant to interact with individuals affected by leprosy, largely due to the misconception of its high contagiousness.

So, their first response is like what? [Is] it even okay to touch them? You know you have to clear their misconceptions. (Dermatology Resident, 28, Interview 3)

This initial hesitation was then addressed by senior dermatologists guiding residents through the process of examining and counseling leprosy patients, fostering confidence and understanding.

So initially the juniors and the doctors which are unaware, they were scared that we will get leprosy by touching, only touching the patient. (Dermatology resident, 32, Int 4)

According to the interviewees, the isolation and quarantining of PAL in general hospitals, even after treatment, continues to foster neglect and hesitancy to touch PAL due to fears of getting infected. Such behavior was noticed to be more prevalent among non-dermatologists and the elder generation of HCW. Some of the nurses, dermatologists and LTs explained to be more open to interactions with PAL:

You know, immediately when they get to know it’s leprosy, they are running for the gloves. They are wearing the mask. I mean, that is totally fine. Like we obviously have to do that, but not in such a hustle as if, you know, that makes the patient feel embarrassed. That shouldn’t be done. (Dermatology Resident, 28, Interview 6)

#### Delay of diagnosis and alternative medicine

According to the interviewed HCW, delay of diagnosis is still very prominent, with PAL presenting only when experiencing severe symptoms, like nerve damage, widespread of hypopigmented patches and loss of sensation (type 1 and 2 reaction). Thus, frequent delay of diagnosis by 10–20 years, exacerbate the effects of leprosy on body and mind, through irreversible disabilities, fueling stigma and discrimination. Furthermore, delay of diagnosis contributes to ongoing transmission among the contacts of infected individuals.

Several reasons appear to be responsible for causing such delay, like misdiagnosis, economic, cultural and social factors. As specified by interviewees, it appeared that GPs, internal medicine specialists and even dermatologists oftentimes failed to recognize symptoms of leprosy, mistaking it for conditions such as psoriasis, tuberculosis, typhoid, urticaria or viral illnesses.

He had 3 episodes, so he visited multiple dermatologists and he showed me the previous prescription. […] Obviously when I saw the prescription, even I was shocked [what] he was prescribed. The others might have also thought it was Urticaria, they misdiagnosed him with that and he was on treatment for Urticaria for the last year. (Dermatology Resident, 28, Interview 6)

As this often leads to wrongful treatment approaches, an LT shared an example, highlighting the dangers of misdiagnoses.

The doctor she was visiting, he asked her to […], apply warm water therapy. And that lady was already like, her hands were anesthetic. And she put her hand on warm water, which was almost boiled, boiled water, and the skin was disappeared, and it was like scar everywhere, burned everywhere. […] It was almost, almost 7 to 8 years delayed. (LT, 33, Interview 20)

To avoid travel, and associated costs, HCW explained that many PAL oftentimes seek care at local doctors and nearby clinics who are less specialized or equipped to provide an adequate diagnosis, further delaying accurate treatment.

[T]hey just go to the doctors, practitioners who are sitting in the nearby clinics who are opening. (LT, 33, Interview 20)

Another contributing factor to the delay of diagnosis was described to be the tendency of PAL to turn to alternative medicine in their search for care. Traditional medicine specialists, homeopathic practitioners or so called “quacks” were frequently visited in the early stages of the disease. HCW portrayed them as individuals that prioritized monetary gain over the patient’s well-being, lacking “actual knowledge”. Treatment such as acupuncture, hijama therapy and cupping therapy, along with the use of Hakimi and Bifakir medication appeared to be widespread, leading to mismanagement of the disease.

They are treating the patients wrong or just treating them for their own satisfaction or for making money. They don’t have much knowledge. (Dermatologist, 30, Interview 5)

Moreover, it was reported that PAL approached spiritual healers, visited shrines and indulged in religious rituals and prayers to get cured.

I have heard stories from women that they go, […] a lake named Mangobi […] and take a bath and next, they believe that the salts and that like water heal their leprosy. Though they think that is a prophetic disease. (Dermatology Resident, 28, Int 12)

This cycle typically continued until prominent patches and significant loss of sensation or disfigurements compelled patients to seek care at a hospital, by which point the disease was usually advanced.

## Discussion

This qualitative study explored the perceptions and experiences of HCW providing care in the field of leprosy, capturing their observations regarding stigma and its interconnected pathways, including how these dynamics influence health behavior. Consequently, perceived and anticipated stigma emerged most prominently, particularly in relation to delayed care-seeking, misinformation, socio-economic discrimination, and exclusion. In contrast, enacted and internalized stigma could only be explored indirectly, and was primarily shaped by PAL reporting on mental health issues, isolation and self-blame. Although stigma was the central analytical focus and guiding framework of this study, the findings were further shaped by Pakistan’s low-endemic context, which highlighted the interdependence between stigma, misinformation and delayed diagnosis (Fig. 3, Supplementary Material 2).

### Study context and leprosy stigma in Pakistan

Pakistan’s low-endemic setting presents a rare epidemiological case, where leprosy was officially declared as eliminated almost thirty years ago, with a prevalence rate below 1 per 10 000 population [13]. Despite a 75% decrease in new adult cases between 2001 and 2023, the mean proportion of new patients presenting with grade 2 disabilities remains high [13, 26]. Furthermore, Pakistan lacks a national leprosy control program. This is in contrast to countries with high-endemic rates, such as India, Indonesia and Tanzania for instance, whose control activities are strengthened by national programs [27–29]. As leprosy remains highly stigmatized, partly due to a widespread of misinformation and limited public awareness, early care-seeking and treatment adherence continues to be jeopardized [30]. A study from Karachi found, that PAL are affected not only physically, through disability and chronic disease, but also mentally, experiencing depression and anxiety stemming from social exclusion, abandonment by family and friends, and difficulties in finding or maintaining employment [31].

### Mitigating stigma by creating awareness

Limited awareness emerged as a key factor in Pakistan’s low-endemic setting, closely intertwined with stigma, at times even outweighing it. In terms of creating more awareness of a disease that has been synonymous with exclusion and discrimination for centuries and now seems to be “dying from the people’s minds”, it is necessary to build on existing awareness activities and to improve active case-finding by detecting new cases as early as possible [26]. This can be achieved by targeting rural areas, Indian and Afghan immigrant communities and the younger generation specifically. In line with that, ongoing projects have shown great success through the facilitation of camps in diverse settings [16], though to assure better cost-effectiveness and a wider reach, participants highlighted the importance of grassroots initiatives. For instance, using social media as a source to spread awareness on early signs and treatment options was suggested by HCW, noting that almost everyone has access to mobile phones or media outlets nowadays. This was found to be effective in a study from Indonesia, where the social media platform Instagram was used to promote knowledge and reduce stigma associated with leprosy [32]. Additionally, digital health tools can support leprosy diagnosis and counseling. Previous research on skin neglected tropical diseases (NTDs) and digital health tools highlighted the potential of portable and offline adapted tools, such as digital diagnostic algorithms and tele-dermatology services, particularly for remote areas. Along with that, digital health tools can assist in streamlining a more cost-effective treatment process for low-endemic settings like Pakistan when combined with rapid diagnostic tests. However, it is important to note that digital health tools should only support HCW and not replace in-person medical care, as more insights are required on the diagnostic accuracy, data security, and user-experience [33]. Rigorous implementation research is needed to evaluate the feasibility and diagnostic accuracy of digital health tools, including tele-dermatology and mobile-based counseling platforms, for leprosy in rural or resource-limited settings. According to LT from this study, another approach that has proven to be effective in reaching more people is to visit communities and PAL on Sundays, when more people are at home instead of at school or work.

### The role of HCW in reducing stigma and supporting treatment adherence

Next to addressing knowledge gaps among the general population, a systematic review showed that knowledge levels and attitudes also appeared to differ between health professions. This is consistent with our study findings, showing that interviewed nurses and GPs have less knowledge about stigma and leprosy when compared to dermatologists and LTs [34].

Alongside, our results highlighted the significance of involving local doctors and alternative medicine specialists by briefing them about early signs to reduce delays, misdiagnoses, and wrong treatment [35]. Beyond accurate recognition and diagnosis, it is essential to ensure a comfortable and positive HCW-PAL relationship. This requires more psychosocial education among HCW within and outside the field of leprosy [36]. HCW need to be taught about the significance they hold in the psychosocial well-being and rehabilitation of PAL. In particular, coping-mechanisms should be tailored to specific needs of affected persons, for instance by respecting their religious beliefs and socio-economic circumstances. Our findings suggested that relationship-building strategies between HCW and PAL emerged as central to supporting treatment adherence and mitigating stigma. A trusting HCW-PAL relationship is therefore not a simple by-product of clinical care but a necessary foundation.

Building on that, other studies from Ethiopia and Nigeria found that the more training HCW received on empathetic communication and psychosocial support, the more positive their attitudes toward PAL became [19, 37]. Beyond formal training, allowing medical students and residents to hear and learn directly from PAL’s stories, may help cultivate empathy among HCW from the outset. With that, embedding practices such as innovative methods of participatory storytelling into HCW training programs has significant potential to strengthen trust-building between HCW and PAL in the Pakistani context [32]. Additionally, HCW must acknowledge PAL’s preferences regarding disclosure of their diagnosis to social networks, especially when approaching people in their homes. Hence, the discrepancy between avoiding stigma by non-disclosure while aiming to tackle stigma through spreading more awareness, becomes evident. As a result, HCW face great complexities, balancing screening, diagnosis and treatment efforts, while simultaneously trying to reduce stigma. Therefore, it is worth considering having a team responsible only for leprosy awareness to relieve HCW of carrying out all responsibilities at the same time.

Building on our findings, it is important to examine HCW-PAL relationships in greater depth to understand how they influence long-term treatment adherence and psychosocial outcomes. Future research is needed to explore whether specific trust-building strategies yield measurable improvements in adherence. Furthermore, this study has identified limited to no stigma towards HCW working in the field of leprosy. According to a comprehensive systematic review from 2022 [34], there remains a knowledge gap on this aspect. While some HCW displayed hesitancy due to fear of contagiousness, which is consistent with six other studies from Indonesia, Ethiopia, Nigeria, South Africa, Thailand and Brazil, our interviewed HCW appeared to feel no stigma towards PAL [19, 37–41]. Other than a study from Nigeria, where 7% believed PAL to be evil doers and 5% assumed them to be witches and wizards [42]. Moreover, three studies from Sri Lanka, Guyana and Ethiopia revealed that HCW felt that PAL should be isolated, although not clear whether for treatment or in their living space [19, 43, 44]. In contrast, our interviewed HCW emphasized the importance of overcoming the separation of PAL from the general population in the hospital setting, indicating there to be no harm when assuring proper protection. The differing perceptions and attitudes of HCW in this study are likely explained by their training at MALC, Pakistan’s primary provider of specialized leprosy care. The low number of new cases and the limited presence of individuals with leprosy-related disabilities and disfigurements may also contribute to reduced stigma among HCW in Pakistan.

### Gender inequalities in leprosy care and stigma

Gender emerged as an important lens to understand social realities of care in Pakistan’s patriarchal context, where societal expectations shape both stigma and access to treatment. Consistent with a systematic review on gender and leprosy-related stigma, this study displayed gender differences linked to leprosy diagnosis, treatment and stigma [45]. In a society like Pakistan, women tend to be treated as inferior to men. Thereby, men carry most economic responsibilities, while women take on domestic duties, caring for their family and home [45]. Moreover, women more commonly face stressors related to violence and constant care responsibilities [45]. As our findings show, such gender inequalities are reinforced in leprosy, with women being described to suffer more, being blamed more, and being less likely to seek care outside of their homes. Facing anticipated stigma thus leads to the concealment of symptoms, which is reinforced by internalized stigma in the form of fear and anxiety, as described by HCW. Similar to studies from India and Vietnam, HCW pointed out that female PAL have shared fears about abandonment from their husband and family in law along with concerns about their physical appearance and how this may affect their chances of finding marriage [46–48]. Men, on the other hand, are described to experience economic distress, as they try to provide financial stability for their families while dealing with leprosy Although men generally have greater access to care than women, they often ignore symptoms and fail to adhere to treatment in order to continue working and fulfill their role as family breadwinners. According to a study from two regions of Nepal, researchers found that women were diagnosed one to two years later than men, while another study from southeastern Nigeria displayed the delay of diagnosis for women to be twice as long as for men [38, 49]. While this study did not identify any gender-related differences in diagnostic delays, our findings suggest a greater diagnostic delay in Pakistan by five to ten years compared to higher-endemic settings. Given this significant delay, it is worth exploring how gender may influence these diagnostic delays in future research endeavors. As the impact of gender norms in a society like Pakistan becomes evident, it is crucial to empower both women and men in the context of their societal roles. Thus, more research is required to inform gender-sensitive approaches, interventions and policies, particularly in low-endemic settings, where there appears to be an even greater delay, associated with more deformity and disability, ultimately enforcing stigma.

### Implications for policy

In line with current global leprosy research priorities, including the Sustainable Development Goals (SDGs) and the World Health Organization (WHO) Global Leprosy Strategy 2021–2030 “Towards zero leprosy” [14, 50], this study contributes to a deeper understanding of leprosy-related stigma in Sindh, Pakistan. Interviews revealed strong confidence in achieving leprosy elimination within the next five to ten years, underscoring a perceived window of opportunity for targeted policy action. Building on these perspectives, several implications for policy can be drawn from the results. Overall, PAL and HCW should be actively involved in decision-making processes shaping stigma-reduction policies and leprosy training initiatives. Training for HCW should be strengthened, particularly with regard to disease transmission and psychosocial aspects, through enhanced peer exchange and support. Moreover, integrating leprosy control into broader dermatology programs, as implemented in Togo, especially for skin-related NTDs, could improve cross-sectoral knowledge, training capacities and cost-effectiveness [26]. Incorporating mental health services into national care plans may further support a more uniform and comprehensive model of care. Finally, securing research funding from diverse sources is essential to ensure that policies remain effective and responsive to the needs of those affected.

### Limitations

This study contains a few limitations that need to be considered. Although most participants were fluent in English, interviews were not conducted in the participant’s native language (Urdu), which may have allowed for more comfort. The sample size was uneven by profession and gender, with more female HCW and dermatologists in residency. The interviewer's distant affiliation with MALC may have introduced social desirability bias, with participants hesitating to disclose negative workplace experiences. However, all interviews were conducted by SCWU as an external researcher. MALC staff and co-authors only facilitated access and were not involved in interviews, though their input helped SCWU understand the local context. Participants did not know the interviewer personally, minimizing potential influence on responses and interpretation. Moreover, interpretation may be influenced by framework adaption and partially inductive coding. Altogether, the categories of perceived and anticipated stigma proved as a good fit, while enacted and internalized stigma dimensions could only be explored from an observer perspective.

## Conclusions

This study highlights the experiences, perceptions, and understandings of HCW working in the field of leprosy in Pakistan. Although great progress has been made, stigma persists, and misinformation and delay of diagnosis increases, due to limited cases, gender roles, religious beliefs, and socio-economic factors.

Trust-building and positive relationships between HCW and PAL emerged as essential for improving treatment adherence and mitigating stigma. Gender dynamics further complicate access to care, with women facing greater barriers to diagnosis and social support, while men often prioritize economic responsibilities over treatment. HCW face complex challenges balancing clinical duties and stigma management, suggesting the value of dedicated awareness teams.

A variety of interventions are required, including greater community outreach through social media and digital health tools; close collaboration with PAL, local doctors, alternative medicine specialists, and religious or social community leaders; strengthened psychosocial training for HCW; and gender empowerment within social roles. Policies should integrate leprosy control into national health strategies, incorporate mental health services, and engage both PAL and HCW in shaping stigma-reduction initiatives.

## Supplementary Information

Below is the link to the electronic supplementary material.

Supplementary Material 1

Supplementary Material 2

Supplementary Material 3

Supplementary Material 4

Supplementary Material 5

## Data Availability

All important data and results are included in this manuscript or supplementary file. To protect the confidentiality of participants, transcripts can be accessed in an anonymized format upon request.

## Abbreviations

PAL: Persons Affected by Leprosy
HCW: Healthcare Worker
MDT: Multi- Drug Therapy
NGO: Non-Governmental Organization
MALC: Marie Adelaide Leprosy Center
ALP: Aid to Leprosy
IDI: In- Depth Individual Interviews
AI: Artificial Intelligence
SDR-PEP: Single-Dose-Rifampicin- Post-Exposure-Prophylaxis
OPD: Outpatient Department
DLQI: Dermatology Life Quality Index Score
LT: Leprosy Technician
SES: Socio-Economic Status
NTD: Neglected Tropical Disease
GP: General Physician
SDG: Sustainable Development Goals
WHO: World Health Organization
