# Supplementary material for: Between stigma, misinformation and delay of diagnosis: healthcare worker’s perspectives on leprosy care in Sindh, Pakistan
Source: BMC Infect Dis. 2026 Feb 2;26:411. doi: 10.1186/s12879-026-12551-z (PMC12924480; doi:10.1186/s12879-026-12551-z)
Supplement: Supplementary file 1 — Supplementary Material 1 [file 12879_2026_12551_MOESM1_ESM.docx]

**Supplementary Material 1:** Fig. 1 Flowchart Study Process
