# Supplementary material for: Between stigma, misinformation and delay of diagnosis: healthcare worker’s perspectives on leprosy care in Sindh, Pakistan
Source: BMC Infect Dis. 2026 Feb 2;26:411. doi: 10.1186/s12879-026-12551-z (PMC12924480; doi:10.1186/s12879-026-12551-z)

**Supplementary Material 2: Fig 3.** Interrelationship Between Stigma, Misinformation, and Delay of Diagnosis


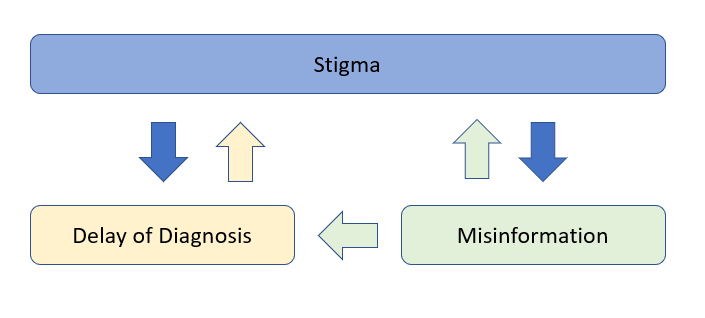

Supplement: Supplementary file 2 — Supplementary Material 2 [file 12879_2026_12551_MOESM2_ESM.docx]
