# Supplementary material for: Between stigma, misinformation and delay of diagnosis: healthcare worker’s perspectives on leprosy care in Sindh, Pakistan
Source: BMC Infect Dis. 2026 Feb 2;26:411. doi: 10.1186/s12879-026-12551-z (PMC12924480; doi:10.1186/s12879-026-12551-z)
Supplement: Supplementary file 3 — Supplementary Material 3 [file 12879_2026_12551_MOESM3_ESM.docx]

**Supplementary Material 3:** Interview Guideline: Medical Doctors specializing in dermatology/in residency

Introduction

1. **Thank you for taking your time to participate in this interview/ study!**
2. **Short Introduction of my person/translator:**

I am Sophie, a 24-year-old researcher with a background in Public Health and Global Health. This project is part of my PhD studies, which I am conducting as research coordinator at MALC in collaboration with the University of Bremen in Germany.

1. **Short explanation of the research project/ aims**

As you have already read in the information document, we are conducting an interview study aiming to learn more about people’s experiences/ perceptions with leprosy and how it impacts their day-to day life and their role in society. By talking to dermatologists in residency, healthcare workers and people affected by leprosy, we strive towards integrating your experiences/opinions/perceptions into existing detection and treatment approaches, or contribute to the development of new, targeted, contextualized interventions to reduce leprosy and the associated stigma.

1. **The interview will approximately take 30-90 minutes/ longer/shorter if necessary.**
2. **Confirmation of consent form and data protection form**

Have you read, understood and signed the consent and information form? Are there any remaining questions concerning the forms that you would like to address or is everything clear?

1. **Ask for approval to record the interview**

As you have already read in the consent form, I would like to record our interview/ conversation for data analysis purposes later on. All recorded information will be later transcribed, pseudonymized and the recordings will then be deleted. I want to double check whether you agree with this procedure or if you would be more comfortable if I only took notes?

1. **Renewed assurance of pseudonymization**

Before we start, I would like to reassure you that everything you are sharing with me today will remain anonymous and won’t be able to be traced back to you.

1. **Voluntary participation**

Naturally I want to confirm that your participation in this interview is voluntary.

- I would like to talk about your experiences with leprosy today. The interview will be structured into 5 parts. I will start by asking you a few general questions, followed by talking about your work experience, your personal experience, local beliefs & perceived stigma and finally I would love to hear whether you had any recommendations for the current leprosy control programs.
- I understand that these are sensitive topics, and some of these questions may bring to mind painful/difficult experiences. You are welcome to share only what you feel comfortable with and can stop or take breaks at any time. If you have difficulties understanding some of the questions, please don’t hesitate to ask any time. I will take some notes for myself so I can better remember what we discussed.

| Theme | Question | Follow-up/Examples |  |
| --- | --- | --- | --- |
| **1. General Information**  **Demographics** | How old are you? |  |  |
|  | Where are you from? | Urban/Rural? |  |
|  | What is your job/education? | What are your daily activities/responsibilities at work? |  |
|  |  | How long have you been working as a dermatologist in residency? When will you finish your residency |  |
|  |  | Why did you choose to specialize in dermatology? |  |
| **2. Work Experience** | Can you tell me a little bit about what you know about leprosy? | What can you tell me about leprosy transmission? How likely do you think it is to get infected with leprosy? |  |
|  |  | What can you tell me about the incubation period? |  |
|  | Can you tell me a little bit about previous experiences you have had with leprosy in a work setting? | Have you ever treated a leprosy patient? If yes, what was that process like? Was it different from what you expected? How did you feel about it? |  |
|  |  | If no, how do you think would that experience be different from treating other skin diseases? |  |
|  |  | Would you talk me through what the process would look like? (E.g.  history taking, clinical examination, differential diagnosis, leprosy classification, and management outline for a new patient) |  |
|  | Would you say that stigma causes a delay of diagnosis? | When at what state of the disease to patients usually come to you for consultation? |  |
|  | Can you tell me a little about what you learned about leprosy in your education? | Have you learned about gender/age differences in leprosy patients? | Partly adapted from PST-toolkit |
|  |  | If treated a patient before, does the process differ from what you have learned? |  |
|  |  | Do you feel well equipped to treat a leprosy patient with that knowledge? If no, would you like to learn more about it? |  |
|  |  | Would you visit a person affected by leprosy in their home for treatment? If no, why not? |  |
|  | If yes: How did the leprosy patients behave around you?  If no: How do you think a leprosy patient would behave around you? | Do you think they felt/ would feel comfortable talking to you about their struggles/hardships? |  |
|  |  | Do you feel like they tend/ would tend to keep information about their illness from you? |  |
|  | Do you think that the associated stigma affects your delivery of care? |  |  |
|  | If yes: What are some beliefs or ideas that patients shared with you about their disease?  If no: Do you think a patient would share beliefs or ideas with you about the disease? | Would you address patient’s beliefs about the disease? If yes, how? If no, why? | Maybe ask about personal faith/religion as coping strategy |
|  |  | Would you address mental health coping strategies that they could engage in? If yes, how, If no, why? |  |
|  | Can you tell me about the views/perceptions of HCWs you know regarding leprosy patients? | Have you witnessed/ heard about positive examples of colleagues that helped to reduce exclusion, discrimination, discomfort? Could you tell me more about such a situation? (e.g., *regarding touching patients; willingness to examine and treatment)* Does it matter whether patients show visible signs of the disease? | *Reminder anonymous* |
|  |  | Have you seen or heard about cases where leprosy patients were made uncomfortable for having leprosy? Could you tell me more about that? |  |
| **3. Personal Experience** | Can you tell me a little bit about your personal experience with leprosy? | Has anyone you know personally have (have had) leprosy? If yes, what is that like for you? Does/did it have an impact on you/your work? |  |
|  | Would you tell your friends/family about working with leprosy patients? | If no, why not? |  |
|  | What do you think as to how other people (family, friends, community etc.) would think about your work with leprosy patients? | Are these positive or negative experiences for you? | Adapted from PST-toolkit |
|  |  | What are common assumptions you encounter? |  |
|  | Do you fear/ Does your family fear that you would get infected with leprosy/infect them? | If yes, why do you think that is? |  |
|  |  | How does that make you feel? |  |
|  |  | If yes, (how) do you address this with them? |  |
|  | Would you want to know if a colleague/ friend/ member in your community was once affected by leprosy? | If yes/no, why? | Adapted from EMIC-CC/SARI   - Did not ask these questions due to social desirability, it did not seem appropriate |
|  | Would you agree to work with/ employ someone that had leprosy? | If yes/no, why? | Adapted from EMIC-CC/SARI   - Did not ask these questions due to social desirability, it did not seem appropriate |
|  | Would you engage with someone that has/had leprosy in public? | e.g., Eat together at the same table, drink from the same glass greet each other/shake hands, etc. | Adapted from EMIC-CC/SARI   - Did not ask these questions due to social desirability, it did not seem appropriate |
|  | Are you married? | If no, do you think your family would allow you to marry someone that has/had leprosy? If no, why?  Could you imagine you met someone that you get along with very well and are attracted to, could you imagine marrying them if you found out that they had leprosy? If no/yes, why?  Would you change your mind if they were completely healed and showed no visible signs of the illness? If yes/no why? | Adapted from EMIC-CC/SARI   - Did not ask these questions due to social desirability, it did not seem appropriate |
|  | Do you have children? | How would you feel if your daughter/son married someone with leprosy? Would it make a difference if they were healed vs. not healed? | Adapted from EMIC-CC/SARI   - Did not ask these questions due to social desirability, it did not seem appropriate |
|  | Are you religious? | If yes, what is your religion? |  |
|  |  | Would you pray next to a leprosy patient in a mosque? If no, why not? |  |
|  |  | Would you open a fast with someone that has/had leprosy? If no, why not? |  |
| **4. Perceived Stigma & Local Beliefs** | Are there any stories/legends/stereotypes about persons affected by leprosy or leprosy in your community? | What kind of stories? Can you tell me one of these stories? | PST-toolkit |
|  |  | Do you agree with that or not, and why? |  |
|  | Do people have the same chance of finding a job if affected by leprosy? | Why not? | Adapted from EMIC-CC/SARI |
|  | Does having/had leprosy affect people’s access to public places/facilities? | Why? | Adapted from EMIC-CC/SARI |
|  | How do people feel around someone affected by leprosy? | Why? | Adapted from EMIC-CC/SARI |
|  | In what way would having leprosy influence someone’s chance to get married? | Why? | Adapted from EMIC-CC/SARI |
|  | Does having/had leprosy influence people’s decision to visit one’s home? | Why? | Adapted from EMIC-CC/SARI |
|  | Do you think people automatically connect leprosy with low SES, poverty, no education etc.? | Do you believe that there is an association? How does this affect the perception of the disease? Do people in a higher caste feel safer? Do you think this association has always been there? |  |
|  | In what way do religious beliefs/and or teaching influence people’s perception of leprosy? | How? |  |
|  | What is stigma to you? What is leprosy related stigma and how is it expressed? | Please elaborate |  |
|  | What does leprosy embody to you? | Please elaborate |  |
| **5. Recommendations** | What is your opinion on the current leprosy control programs/treatment? | What do you think works well for patients and you? |  |
|  |  | Do you believe the current treatment/ diagnosis process should stay as it is? Change? |  |
|  | Do you think HCWs or persons affected should receive more support by society/politics etc.? | What type of support would be useful to have? |  |
|  | How realistic do you think it is to eliminate this disease within the next 5-10 years? | Why? What would need to happen? |  |
|  |  | Why do you think it hasn’t happened? |  |
|  | Is there anything you would like to say to your community/ colleagues/ family/HCWs about leprosy? | Do you feel you are not able to say it openly to them? If yes, why? | *Reminder: anonymous* |
| **6. Review/Closing** | Is there anything else you would like to add? |  |  |
|  | Do you have any remaining questions? |  |  |
|  | Thank you for your time and participation. The information that you have shared will greatly contribute to the research project. | Renewed assurance of pseudonymization |  |

Outlook

- Information about what is going to happen with the collected interview data next
  - Data will be transcribed, pseudonymized, recording will be deleted.

Disclaimer Information on Leprosy Knowledge

Interview Guideline: Nurses, leprosy technicians

| Theme | Question | Follow-up/Examples |  |
| --- | --- | --- | --- |
| **1. General Information** | How old are you? |  |  |
|  | Where are you from? | Urban/Rural? |  |
|  | What is your job/education? | What are your daily activities/responsibilities at work? |  |
|  |  | How long have you been working as a HCW in the field of leprosy? |  |
|  |  | Why did you choose to work in this field? |  |
| **2. Work Experience** | Can you tell me a little bit about what you know about leprosy? | What can you tell me about leprosy transmission? How likely do you think it is to get infected with leprosy? |  |
|  |  | What can you tell me about the incubation period? |  |
|  | Can you tell me about a (recent) situation with a PAL that stuck to your mind/ was particularly interesting/challenging? |  |  |
|  | Can you tell me a little bit about what the process of working with a PAL is like in general? | Can you tell me a little about what you learned about leprosy in your education? Does the process differ from what you have learned? | Partly adapted from PST – toolkit |
|  |  | How commonly do you work with a PAL? |  |
|  |  | Where do you work with the PALs Is it common to conduct house visits for severely ill PALs? If yes, would you visit them in their home for treatment? If no, why not? |  |
|  |  | Does it make a difference whether someone has visible signs of leprosy or not? |  |
|  |  | How does the whole process make you feel? |  |
|  | When at what state of the disease to PALs usually come to you? Who are they referred by? | Would you say that stigma causes a delay of diagnosis? |  |
|  | How do PALs behave around you? | Do you think they feel comfortable talking to you about their struggles/hardships? |  |
|  |  | Do you feel like they tend to keep information about their illness from you? |  |
|  | What are some beliefs or ideas that PALs share with you about their disease? | Do you address PALs beliefs about the disease? If yes, how? If no, why? |  |
|  |  | Do you address mental health coping strategies that they could engage in? If yes, how, If no, why? |  |
|  | Do you see more female/male/child PALs? | Can you observe a difference in behavior between gender/age? If yes, in what way? |  |
|  |  | If you see more males/females why do you think that is? |  |
|  |  | Is there a difference in treatment between males and females? |  |
|  | How old are your patients usually? |  |  |
|  | Where do your patients usually come from? | Do you think people automatically connect leprosy with low SES, poverty, no education etc.? |  |
|  | Can you tell me about the views/perceptions of HCWs you know regarding PALs | Have you witnessed positive examples of colleagues that helped to reduce exclusion, discrimination, discomfort? Could you tell me more about such a situation? | *Reminder: Anonymous* |
|  |  | Have you seen or heard about cases where PALs were made uncomfortable for having leprosy? Could you tell me more about that? |  |
| **3. Personal Experience** | Can you tell me a little bit about your personal experience with leprosy? | Has anyone you know personally have (have had) leprosy? If yes, what is that like for you? Does/did it have an impact on you/your work? |  |
|  | Do you tell your friends/family about working with leprosy patients? | If no, why not? |  |
|  | According to you experience how do other people (family, friends, community etc.) think about your work with PALs? | Are these positive or negative experiences for you? | Adapted from PST-toolkit |
|  |  | What are common assumptions you encounter? |  |
|  | Do you fear/ Does your family fear that you will get infected with leprosy/infect them? | If yes, why do you think that is? |  |
|  |  | How does that make you feel? |  |
|  |  | If yes, (how) do you address this with them? |  |
|  | Would you agree to work with/ employ someone that had leprosy? | If yes/no, why? | Adapted from EMIC-CC/SARI   - Did not ask these questions due to social desirability, it did not seem appropriate |
|  | Would you engage with someone that has/had leprosy in public? | e.g., Eat together at the same table, drink from the same glass greet each other/shake hands, etc. | Adapted from EMIC-CC/SARI   - Did not ask these questions due to social desirability, it did not seem appropriate |
|  | Are you religious? | If yes, what is your religion? | Used for demographics |
|  |  | Would you pray next to a leprosy patient in a mosque? If no, why not? | - Did not ask these questions due to social desirability, it did not seem appropriate |
|  |  | Would you open a fast with someone that has/had leprosy? If no, why not? | - Did not ask these questions due to social desirability, it did not seem appropriate |
| **4. Perceived Stigma & Local Beliefs** | Are there any stories/legends/stereotypes about persons affected by leprosy or leprosy in your community? | What kind of stories? Can you tell me one of these stories? | PST-toolkit |
|  |  | Do you agree with that or not, and why? |  |
|  | Do people have the same chance of finding a job if affected by leprosy? | Why not? | Adapted from EMIC-CC/SARI |
|  | Does having/had leprosy affect people’s access to public places/facilities? | Why? | Adapted from EMIC-CC/SARI |
|  | How do people feel around someone affected by leprosy? | Why? | Adapted from EMIC-CC/SARI |
|  | In what way would having leprosy influence someone’s chance to get married? | Why? | Adapted from EMIC-CC/SARI |
|  | Does having/had leprosy influence people’s decision to visit one’s home? | Why? | Adapted from EMIC-CC/SARI |
|  | Do you think people automatically connect leprosy with low SES, poverty, no education etc.? | Do you believe that there is an association? How does this affect the perception of the disease? Do people in a higher caste feel safer? Do you think this association has always been there? |  |
|  | In what way do religious beliefs/and or teaching influence people’s perception of leprosy? | How? |  |
|  | What is stigma to you? | Please elaborate |  |
|  | What does leprosy embody to you? | Please elaborate |  |
| **5. Recommendations** | What is your opinion on the current leprosy control programs/treatment? | What do you think works well for PALs and you? |  |
|  |  | Do you believe the current treatment/ diagnosis process should stay as it is? Change? |  |
|  | Do you think HCWs or persons affected should receive more support by society/politics etc.? | What type of support would be useful to have? |  |
|  | How realistic do you think it is to eliminate this disease within the next 5-10 years? | Why do you think it hasn’t happened? |  |
|  | Is there anything you would like to say to your community/ colleagues/ family/HCWs about leprosy? | Do you feel you are not able to say it openly to them? If yes, why? | *Reminder:anonymous* |
| **6. Review/Closing** | Brief summary of the interview |  |  |
|  | Is there anything else you would like to add? |  |  |
|  | Do you have any remaining questions? |  |  |
|  | Thank you for your time and participation. The information that you have shared will greatly contribute to the research project. | Renewed assurance of pseudonymization |  |

Outlook
