# Supplementary material for: Between stigma, misinformation and delay of diagnosis: healthcare worker’s perspectives on leprosy care in Sindh, Pakistan
Source: BMC Infect Dis. 2026 Feb 2;26:411. doi: 10.1186/s12879-026-12551-z (PMC12924480; doi:10.1186/s12879-026-12551-z)
Supplement: Supplementary file 4 — Supplementary Material 4 [file 12879_2026_12551_MOESM4_ESM.docx]

*Research project on leprosy-related stigma among people affected by leprosy and health care workers in Sindh, Pakistan*

**Declaration of consent**

I have read the information document and was informed verbally by the responsible person about the aim and the course of the study as well as about the risks in a detailed and understandable manner. I had the opportunity to ask questions during the informed consent discussion. All my questions were answered to my satisfaction. I voluntarily agree to participate in the study. I had sufficient time to make my decision. I have received a copy of the information document and the declaration of consent.

**Data protection**

**I am aware that personal data will be processed in this study.** **The processing of the data is carried out in accordance with legal provisions and requires the following declaration of consent in accordance with Art. 6 para. 1 lit. a of the General Data Protection Regulation:**

**I have been informed and voluntarily agree that my data collected in the study, will be recorded and evaluated in pseudonymized form for the purposes described in the information ldocument.** **Third parties will not have access to personal data.** **If necessary, data will only be passed on to other research institutions in anonymized form, possibly also to countries with lower data protection requirements than in the European Union.** **My name will also not be mentioned when the results of the study are published.** **The personal data will be anonymized as soon as this is possible according to the purpose of the research.** **The data will be stored for 5 years after completion of the study. I am aware that this consent can be revoked at any time in writing or verbally without giving reasons and without any disadvantages for me.** **This does not affect the legality of the data processing carried out up to the point of withdrawal.** **In this case, I can decide whether the data collected from me should be deleted or may continue to be used for the purposes of the study.**

**I have been assured that the handling of my data and the study results complies with the provisions of the Bremen Data Protection Act.**

Place, Date Surname, First name of participant

(in block letters)

Signature of the Participant

**Informing person**

The participant was informed by me during the interview about the aim and procedure of the study and about the risks. I have given the participant a copy of the information document and the declaration of consent. As the project leader and scientist responsible for this research project, I will ensure that all requirements of the Bremen Data Protection Act are taken into account and that the data will not be used for commercial purposes, but only for the purpose of the study.

Place, Date Surname, First name of the informing person

(in block letters)

Signature of the informing person
