# Supplementary material for: Between stigma, misinformation and delay of diagnosis: healthcare worker’s perspectives on leprosy care in Sindh, Pakistan
Source: BMC Infect Dis. 2026 Feb 2;26:411. doi: 10.1186/s12879-026-12551-z (PMC12924480; doi:10.1186/s12879-026-12551-z)
Supplement: Supplementary file 5 — Supplementary Material 5 [file 12879_2026_12551_MOESM5_ESM.docx]

*Research project on leprosy-related stigmatization among people affected by leprosy and health care workers in Sindh, Pakistan*

**Information Document for Participants**

Dear Sir or Madam,

With this letter we invite you to participate in the above mentioned study. Please read the following information carefully. You can then decide whether you wish to participate or not. Allow yourself sufficient time and ask the study staff any questions that are important to you.

The study is funded by the Marie Adelaide Leprosy Center (MALC) through the German Leprosy and Tuberculosis Relief Association (DAHW). The principal investigator of this study, Sophie Stützle (now Unterkircher), is research coordinator at MALC and a Dr. Public Health candidate at the University of Bremen, Germany.

**What is the aim of the study?**

The aim of the research project is to investigate the stigma associated with leprosy (for example, embodied in discrimination, exclusion, withholding of social acceptance, negative evaluation and prejudice) in Pakistan by examining the causes and perceptions of people affected by leprosy, healthcare workers and dermatologists in residency. This interview study will help to increase knowledge about the role of stigma in Sindh. This knowledge can be integrated into existing detection and treatment approaches, or contribute to the development of new, targeted, contextualized interventions to reduce stigma.

**How will the study work?**

As part of the study, we would like to conduct a guided interview with you lasting approximately 30-90 minutes. In this interview, experiences and perspectives regarding leprosy-related stigma will be discussed (e.g., work experiences, personal experiences, perceived/societal experiences, recommendations). Depending on your preference, the interview can take place via a video conferencing platform or by telephone. The interviews will be conducted by the principal investigator in English and, if needed, together with an interpreter in Urdu.

Following written consent, the interview will be recorded using a dictation machine. The recording is used for the subsequent interview transcription and facilitates interview management and data analysis. The recorded interviews are transcribed as full texts (noscribe) and pseudonymized. If a recording is not desired, a detailed protocol can be kept instead, which is also pseudonymized for the analysis.

**Do I have a personal benefit?**

The information obtained is used for scientific purposes. There are neither advantages nor disadvantages from your participation or your answers. If you are interested, we will be happy to send you the results report of the study.

**Information on data protection**

The data protection regulations are complied with. The data important for the study will also be stored and evaluated in pseudonymized form. "Pseudonymization" is the processing of personal data in such a way that the personal data can no longer be assigned to a specific data subject without the use of additional information ("keys"). This additional information is stored separately and is subject to technical and organizational measures that ensure that the personal data cannot be assigned to an identified or identifiable natural person. The study management will take all reasonable steps to ensure the protection of your data in accordance with the data protection standards of the European Union. The data is secured against unauthorized access. Decryption will only take place in the event of withdrawal from the study for the purpose of data destruction. As soon as it is possible for research purposes, the personal data will be anonymized. "Anonymization" is the modification of personal data in such a way that the person concerned can no longer be identified or can only be identified with a disproportionate amount of cost or time. On request, data collected during the study may be passed on to other research institutions, but only in anonymized form. Under certain circumstances, these institutions may be located in countries with lower data protection standards than the European Union. The data collected during the study will be kept for 5 years after completion of the study and then deleted. The data will only be used for the purposes of this study.

You have the right to request information from the controller (see below) about the personal data stored about you. You can also request the correction of inaccurate data and the deletion of the data or the restriction of its processing.

The person responsible for the study-related collection of personal data is:

Sophie Stützle, M.Sc. (now Unterkircher)

Research Coordinator MALC

Marie Adelaide Leprosy Center,

Mariam Manzil, A.M. 21

Off Shahrah-e-Liaquat, P.O. Box No. 8666

Saddar Karachi, 74400 Pakistan

Business e-mail address: [sophie.stuetzle@web.de](mailto:sophie.stuetzle@web.de)

In the event of unlawful data processing and similar matters, you have the right to lodge a complaint at any time using the following contact details

German Leprosy and Tuberculosis Relief Association (DAHW)

Advisor Humanitarian Aid and Safeguarding

Mr. Imran Khan

E-mail: [imran.khan@web.de](mailto:imran.khan@web.de)

**Voluntary participation/withdrawal**

Participation in the study is voluntary. If you wish to participate, we ask you to sign the enclosed declaration of consent. You can withdraw this consent at any time in writing or verbally without giving reasons and without incurring any disadvantages. If you wish to withdraw your consent, please contact the study management. If you withdraw your consent, you can decide whether the data collected from you for the study should be deleted and the recordings made destroyed or may continue to be used for the purposes of the study. Even if you initially consent to further use, you can change your mind at a later date and request the deletion of the data or recordings; please also contact the study management for this. Please note that data that has already been included in scientific evaluations or data/recordings that have already been anonymized can no longer be deleted at your request.

**Will I incur any costs by participating?** **Will I receive any payment or reimbursement of expenses?**

Participation in the study is free of charge for you. However, you will not receive any payment. You will be reimbursed for travel expenses.

**Further information**

For further information as well as for information on general results and the outcome of the study, please contact Ms. Sophie Stützle (now Unterkircher) (e-mail: [sophie.stuetzle@web.de](mailto:sophie.stuetzle@web.de)) as head of the study.

**We would be very grateful for your participation in the research project!**
